# Topical Diagnosis and Determination of the Primary Hyperaldosteronism Variant

**DOI:** 10.25122/jml-2019-0072

**Published:** 2019

**Authors:** Viktor O. Shidlovskyi, Olexandr V. Shidlovskyi, Oleksandr A. Tovkai, Michael I. Sheremet, Vitaliy V. Maksymyuk, Volodimir V. Tarabanchuk, Shved M. Ivanovych, Mykolaivna S. Heryak, Mykhailovych S. Andreychyn, Igorivna I. Hanberher, Antonina A. Piddubna

**Affiliations:** 1. Surgery Department, I. Horbachevsky Ternopil National Medical University, Ternopil, Ukraine; 2. Ukrainian Scientific and Practical Center of Endocrine Surgery, Endocrine and Tissue Transplantation of the Ministry of Health of Ukraine, Kyiv, Ukraine; 3. First Surgery Department, Bukovinian State Medical University, Chernivtsi, Ukraine; 4. Department of First Emergency Medical Aid and Emergency Medical Treatment, Ternopil, Ukraine; 5. Second Department of Obstetrics and Gynecology, I. Horbachevsky Ternopil National Medical University, Ternopil, Ukraine; 6. Department of Propaedeutic of Internal Medicine and Phthisiology, I. Horbachevsky Ternopil National Medical University, Ternopil, Ukraine; 7. Department of Clinical Immunology, Allergology and Endocrinology, Bukovinian State Medical University, Chernivtsi, Ukraine

**Keywords:** primary hyperaldosteronism, laboratory diagnosis, influencing factors on its results, ARR - aldosterone-to-renin ratio, PHA - primary hyperaldosteronism

## Abstract

Laboratory diagnosis of primary hyperaldosteronism is based on determining blood levels of aldosterone, renin on request, potassium, and sodium. The results of these studies are significantly influenced by drugs, preparation for the study and blood collection methods, age, gender, and concomitant diseases. The work analyzes the factors influencing the results of the study of aldosterone and identifies the main ways of their exclusion at each stage of the diagnosis. Their neglecting is the determining factor in obtaining false results, diagnostic errors, the selection of ill-treatment tactics, and inadequate treatment. All these diagnostic problems are covered in a variety of ways in the review, which is based on the analysis of results of individual authors’ research and practical and clinical recommendations from leading world endocrinological associations.

Results of laboratory diagnostics of PHA depend on the influence of many factors. Among them, it is essential to use different medication drugs, the rules for preparing for the study, and the method of conducting it. In assessing the results of research, it is necessary to take into account not only the indicators of the level of aldosterone in the blood but also the features of the clinical course of the disease, its compliance to the drug therapy, age, and gender of the patients.

## Introduction

Laboratory diagnosis of primary hyperaldosteronism (PHA) is based on the determination of blood levels of aldosterone and renin and is carried out at all three stages of the diagnostic process. The task of the laboratory diagnosis of PHA in the first stage of screening among patients with arterial hypertension and suspicion of hyperaldosteronism is to establish its presence. In the second stage, primary hyperaldosteronism is confirmed using the tests based on the concept that in PHA, aldosterone is secreted in an unregulated way and therefore not suppressed by normal physiological mechanisms. In the third stage, with the help of laboratory tests, the questions concerning the differentiation of PHA subtypes according to the results of the study of the content of aldosterone in blood samples taken selectively from the veins of the adrenal glands are solved [1-3].

However, in the laboratory diagnosis of PHA, there are two essential problems: the evaluation of research results and observance of the protocol of research and the rules of preparation for it. The essence of the first problem is that today, before the evaluation of aldosterone and renin levels in the blood, there are no commonly accepted approaches. Different methods of research (radioimmunological or immunoenzymometric analysis) and units of measurement are used. Thus, the aldosterone level of 1 ng/dl corresponds to 27.7 pmol/l in units of the SI system; plasma renin activity of 1 ng/ml/hr corresponds to 12.8 pmol/l/min in units of the SI system, and direct renin concentration is approximately 8.2 mU/l or 5.2 ng/l in conventional units. In the publications devoted to the diagnosis and treatment of PHA, the results of the research, conclusions, and recommendations are based on indicators performed on different methods presented in different units of measurement, which significantly impedes the analysis, comparison, and evaluation of the results presented by the authors [4-6].

Most authors use the following indicators: for the level of aldosterone – in the range of 20-40 ng/dl, for the activity of plasma renin – within 68-135 ng/ml/hr in venous blood, taken in the morning, with the patient in a sitting position.

Due to the lack of standard approaches to detection methods and measurement units, there is significant variability in the determination of the diagnostic value of the aldosterone-to-renin ratio (ARR) in primary hyperaldosteronism.

Some scientists believe that in order to positively diagnose PHA, apart from the increased value of ARR as a criterion for diagnosis, it is necessary to increase the aldosterone level> 15 ng/dl or 416 pmol/l [7]. Other researchers point out that it is necessary to avoid the formal significance of the upper limit of aldosterone norm, but one must understand that there is a higher probability of a false positive result of the aldosterone-to-renin ratio at low levels of renin [8]. An example of such a result is the inappropriateness of considering the formal value of the upper limit of the aldosterone norm as a rigorous diagnostic criterion for primary hyperaldosteronism. Thus, in 36% of 74 patients diagnosed with primary hyperaldosteronism, according to screening results, ARR was greater than 30 (>100), with aldosterone levels lower than 15 ng/dl (<416 pmol/l).

The diagnosis of primary hyperaldosteronism in these patients was confirmed by the lack of aldosterone suppression in a fludrocortisone test, in 4 out of 21 patients, according to a blood test selectively taken from the veins of the adrenal glands; unilateral hyperproduction of aldosterone was found and then surgically treated [9]. In another study, aldosterone levels in the range of 9-16 ng/dl (250-440 pmol/l) were detected in 16 out of 37 patients with suspicion of PHA, confirmed by an intravenous infusion of a physiological solution [10]. Therefore, taking into account these data, it should be considered that the use of an obligatory limit of aldosterone of more than 15 ng/dl for the diagnosis of PHA can be a cause of diagnostic mistakes.

Thus, ambiguous expert opinions and controversial literature data, the variability of laboratory parameters for aldosterone and renin levels, which depend on the applied method of blood draw, laboratory features, the impact of drugs, age, and others require to refrain from rigorous recommendations regarding the diagnostic value of the aldosterone-to-renin ratio. It is essential to provide doctors with comprehensive information on the advantages and disadvantages of the techniques, factors that affect the outcome of the aldosterone-to-renin ratio, and the ability to individually interpret the results of the studies conducted.

Today, ARR is the most reliable and accessible method for screening primary hyperaldosteronism. Despite the identified shortcomings in research on the diagnostic value of ARR, numerous studies confirm its diagnostic advantage over individually applied methods for determining the level of aldosterone or aldosterone and potassium (low sensitivity of both indicators) and renin (low specificity) [11].

The second problem is the dependence of the aldosterone levels and blood renin and implicitly the ARR on the strict observance of the protocol for conducting the study and the rules for its preparation [5]. We consider it expedient to take them into account. They involve:

1. Prior to examining the aldosterone level, it is necessary to determine the content of potassium in the blood. The research should be conducted against the background of normokalemia because hypokalemia inhibits the synthesis of aldosterone and will cause false results;
2. The day before the study, the patient should not restrict the consumption of kitchen salt;
3. Drugs that significantly affect ARR should be discontinued – diuretics, including spironolactone, eplerenone, triamterene, amiloride, licorice products, and take other drugs that reduce pressure (Table 1);
4. If the result of ARR after discontinuing the intake of these drugs is not diagnostically significant and if hypertension can be controlled with drugs that have a minimal effect on the level of aldosterone, it is necessary to cancel at least for two weeks other medication that may affect the level of aldosterone-to-renin ratio.

**Table 1:** Hypotensive drugs with a minimal effect on ARR.

Drug	Class	Dosage	Special warnings and precautions for use
Verapamil, prolonged form	Non-dihydropyridine - new calcium channel blocker	90–120 mg twice a day	Used alone or in combination with other drugs
Hydralazin	Vasodilator	10.0–12.5 mg twice a day with titration to an effective dose	Prescribed after verapamil as a stabilizer for reflex tachycardia
Doxazosin	Alpha1 adrenergic blockers	1–2 mg once a day with titration dose to desired clinical effect	Prescribed to control postural hypotension
Terazosin	Alpha1 adrenergic blockers	1–2 mg once a day with titration dose to desired clinical effect	Prescribed to control postural hypotension

The conditions for blood drawing in order to study the aldosterone level, active plasma renin, the direct concentration of renin and potassium are also clearly regulated [12]. They include:

1. Blood samples should be collected in the morning after the patient stayed in a vertical position for at least 2 hours before the study (vertical position – to walk, stand, sit). Immediately before blood collection, one needs to sit for 10-15 minutes;
2. During blood-taking, one should avoid squeezing the hand into a fist, and the collection should be carried out not earlier than 5 seconds after the tourniquet is removed;
3. It is necessary to separate the plasma for research without delay and not later than 30 minutes after blood collection, at the same time keeping the test tube at room temperature and not in an ice bath, as cold increases the content of active plasma renin. In the case of a waiting study, the plasma component should be rapidly frozen;
4. The presence of hemolysis or a blood clot is not allowed, and such cases require repeated blood-drawing [5].

When conducting trials and tests to determine the level of aldosterone and the activity of plasma renin one should take into account some factors that can potentially affect the results of research and choice of therapeutic tactics and create conditions for obtaining false positive or false negative results (Table 2) [13].

**Table 2:** Factors that can affect ARR and cause false positive and false negative results.

Factor	Effect on aldosterone level	Effect on renin level	Effect on the aldosterone-to-renin ratio
Drugs			
**β- adrenoblockers**	↓	↓↓	↑
**Central α2 antagonists**	↓	↓↓	↑
**nonsteroidal antiinflammatory drugs**	↓	↓↓	↑
**Calcium excreting diuretics**	→↑↑	↑↑	↓
**Calcium preserving diuretics**		↑↑	↓
**ACE inhibitor**	↓		↓
**Angiotensin receptor blockers II**	↓	↑↑	↓
**Dihydropyridazine**	→↑	↓↑	↓
**Renin inhibitors**	↓	↓↑*	↓
**Potassium level**			
**Hypokalaemia**	↓	→↑	↓
**Loading**	↑	→↑	↑
**Kitchen salt**			
**Consumption restriction**	↑	↑↑	↓
**Loading**	↓	↓↓	↑
**Other conditions**			
**Older age**	↓	↓↓	↑
**Renal insufficiency**	→	↓	↑
**Pregnancy**	↑	↑↑	↓
**Renovascular hypertension**	↑	↑↑	↓

Notes: * – renin inhibitors reduce the plasma renin activity, but increase direct renin concentration, which may lead to erroneously positive ARR results for renin, measured by plasma renin activity, and erroneously negative results for renin determined by direct plasma renin; ACE – an angiotensin-converting enzyme; ↓ – reduces the level; ↓↓ – significantly reduces the level; ↑ – increases the level; ↑↑ – significantly increases the level; → – does not affect the factor; →↑ – does not affect the factor, or slightly increases; ↓↑ – can reduce or increase; → ↑↑ – does not change, or significantly increases.

## Discussion

Such factors include, in particular, the use of nonsteroidal anti-inflammatory and contraceptive drugs. Anti-inflammatory nonsteroidal drugs reduce potassium excretion, leading to stimulation of the synthesis of aldosterone and a further increase in the aldosterone-to-renin ratio. Progesterone and progestogenic drugs, such as mineralocorticoid receptor antagonists, can induce natriuresis and the synthesis of renin and aldosterone [14]. Non-potassium sparing diuretics (thiazides) increase potassium losses in the kidneys, thereby reducing the level of potassium in the plasma, leading to a decrease in the aldosterone secretion. Potassium-sparing diuretics (spironolactone, eplerenone, and amiloride) stimulate the synthesis of renin [15]. Selective calcium antagonists also increase the plasma renin activity in the treatment. This happens, presumably, through reflexive sympathetic stimulation due to the lowering of blood pressure. These drugs may also interfere with the production of aldosterone because they block the intracellular, calcium-dependent stages of its biosynthesis. Antidepressants at long-term intake are also able to influence the aldosterone-to-renin ratio and distort the outcome of the study; blood levels of aldosterone, active, and direct plasma renin increase in case of their usage [16].

Limited or excessive consumption of kitchen salt affects the ARR index. Hyponatremia stimulates the synthesis of renin and causes a decrease in the ratio, while high levels of sodium in the blood profoundly inhibit the synthesis of renin and cause an increase in ARR [17].

Premenopausal women are advised to detect active plasma renin within the first nine days of menstruation through the effects of sex steroids [18].

The time of day of blood collection for research is influenced by ARR. Given the circadian rhythm of the synthesis of hormones, the level of ARR in the blood increases more often in the morning than in the afternoon. Consequently, in the blood taken in the daytime, mistakenly negative results are possible [19].

Interpretation of results of the first stage of screening in patients with suspected PHA should be conducted taking into account the medical and family history, the severity of arterial hypertension, its resistance to the treatment by antihypertensive drugs, the level of aldosterone in the blood, and the presence of electrolyte disturbances (potassium, sodium, magnesium) [20]. Also, in doubtful cases, one should not rely on only one ARR study; if necessary, it should be repeatedly checked for a long time before making a medical conclusion, especially if the indicator is questionable [21]. In determining the level of aldosterone in ng/dl and the active plasma renin in ng/ml/hr, the ARR of more than 20-25 indicates a high probability of the presence of PHA. When the aldosterone level is measured in pmol/l, the ARR greater than 900 also indicates the benefit of this disease [22].

Physiologically healthy individuals have aldosterone levels of less than 15 ng/dl. However, since the secretion of aldosterone varies depending on the time of day, physical activity, body position change, and others, the negative and positive predictive values of one random aldosterone level are limited. It should also be taken into account that the aldosterone level test has a high sensitivity (95%) and a low specificity (75%). The low specificity of aldosterone levels is due to a significant number of cases of false-positive diagnosis of PHA. In case of inconsistency of the obtained ARR index (less than 20), the clinical follow-up data of the patient recommend a re-examination that is prolonged in time.

The critical issue of early diagnosis of PHA is to establish the presence of autonomous, independent of the influence of the renin-angiotensin-aldosterone system of aldosterone secretion. It is solved utilizing loading tests, which is the essence of the second stage of the diagnosis of PHA.

Loading tests are based on the concept according to which, in PHA, aldosterone is secreted in an unregulated way and not suppressed by physiological mechanisms. Contraindications to their implementation are the aldosterone-to-renin ratio in plasma above 50, the combination of spontaneous hypokalemia and aldosterone levels greater than 20 ng/dl (550 pmol/l), low or not determined, direct plasma renin or its activity [23].

The diagnosis of PHA is confirmed or excluded by loading tests with kitchen salt, fludrocortisone, or captopril. None of them is sufficiently reliable and can not be recognized as a priority. Significant variability in data on their sensitivity, specificity, and reliability allows choosing a specific method depending on the financial capabilities and patient’s compliance, the features of the laboratory, the benefits of one of the tests, or several of them by particular doctors.

Loading tests with sodium chloride can be performed using the protocol of intravenous administration of saline solution or that of oral intake of salt. Assertive tests based on saline loading, due to their simplicity and reliability, low economic costs, are applied most widely.

### Protocol for intravenous administration of a saline solution

The principle is based on the fact that a sharp increase in the volume of circulating blood and content of sodium in plasma at the expense of the physiological solution causes the inhibition of the aldosterone secretion. In the case of PHA, this inhibition is not observed.

Prior to testing, if possible, hypotensive therapy should be canceled two weeks before the study. During one hour before the test and throughout the test, the examined patient should lie on the back. At 7:30, they set up a venous catheter and take blood to determine basal concentrations of aldosterone and potassium in serum. At 8:00, an infusion of 0.9% NaCl should begin. Infusion duration – 4 hours, speed – 500 ml/hour, total volume – 2 l. After the infusion, blood is collected in order to determine the concentrations of aldosterone and potassium in the serum.

### Evaluation of results

Typically, the concentration of aldosterone in blood serum is reduced by 50–80% and does not exceed 5 ng/dl. The test is not informative – the concentration of aldosterone in the range of 5–10 ng/dl. A high probability of primary hyperaldosteronism is the concentration of aldosterone more than 10 ng/dl.

### Precautions

The study is contraindicated in cardiovascular diseases and renal insufficiency. An increase in the volume of circulating blood can cause severe hypokalemia, so two years after the start of the infusion, it is recommended to determine the concentration of potassium in the serum and to take measures to reduce it.

The infusion test of the physiological solution has a sensitivity of 83–88%. It is believed that the sensitivity of the test increases when it is conducted in a sitting position.

### The protocol for oral intake of salt

The test involves a daily intake of at least 10–12 g of sodium chloride for at least 5 days before the test under the control of daily excretion of sodium under normokalemia. The test can be performed in outpatient conditions. It is necessary to add 10–12 g of kitchen salt to daily ration and follow this diet for 5 days. On the 5^th^ day, daily urine is collected to determine the levels of sodium and aldosterone. In the case of hypokalemia, the drugs of potassium are prescribed additionally.

Evaluation of results. The daily excretion of sodium should not exceed 250 μV, and the aldosterone content in daily urine should be less than 6 μg/day. With a content up to 10 μg/day, the diagnosis of PHA is highly doubtful. In the event when the aldosterone level exceeds 12 μg/day, it is beyond doubt [23].

### Suppressive test with fludrocortisone

If possible, antihypertensive therapy should be aborted two weeks before the study. Hospitalization is required, and the consumption of salt is not limited. Basal levels of aldosterone, active plasma renin and cortisol content are determined in blood serum. For three days, patients take fludrocortisone at a dose of 0.2 mg 3 times a day. On the morning of the third day, the levels of aldosterone, renin, and cortisol are determined. If it is planned to determine the content of aldosterone in urine, it is collected during the third day of fludrocortisone consumption.

### Evaluation of results

Typically, after the administration of fludrocortisone, the concentration of aldosterone in serum is lower than 3 ng/dl, and its excretion with urine is less than 20 μg/day. In the case of PHA, high aldosterone indices in blood serum or urine are determined, which are not decreased during the test. The test with fludrocortisone is contraindicated in patients with hypokalemia, cardiovascular disease, and renal insufficiency. The complexity of accomplishment is its disadvantage. Besides, it requires hospitalization of the patient, which increases the duration and cost of the diagnostic examination. Test adherents emphasize its safety and superior sensitivity in comparison with other research methods [24].

### Captopril test

The patient should take 25 mg of captopril in 1 hour after morning waking. Before taking it and 2 hours later, a blood sample is taken to determine the levels of aldosterone, cortisol, and plasma renin activity. During the test, the patient should sit down. Usually, after taking captopril, the aldosterone content is reduced by more than 30% from the baseline. The test is considered positive if the level of aldosterone does not decrease after its completion. The disadvantage of the test is the presence of a large number of false-positive and false-negative results, so it is not acceptable as a highly specific diagnostic test.

At the third stage (topical and differential diagnosis of subtypes of PHA), the main task of laboratory research is to distinguish between sporadic (one-and two-way adrenal lesions) and family forms. For this purpose, the levels of aldosterone (if necessary – cortisol and renin) in the venous blood, obtained separately from each adrenal gland, are studied [25]. Determination of the aldosterone level in blood samples selectively collected from the veins of the adrenal glands is a gold standard of diagnosis to clarify the one- or two-way nature of secretion of aldosterone in patients with primary hyperaldosteronism [26]. Also, the lateralization of the aldosterone hyperproduction source is significant for choosing an adequate method of treating PHA.

In case of suspicion of family hyperaldosteronism type I, an adrenal suppression test for dexamethasone is performed. Dexamethasone is prescribed at a dose of 0.5 mg every 6 hours for 48 or 72 hours, then plasma aldosterone levels are determined. Reducing its level below 4 ng/dl confirms the diagnosis of family hyperaldosteronism type I [27]. It is precisely in the case of family hyperaldosteronism type I, on the background of small doses (1–2 mg per day) of dexamethasone, there is a temporary decrease in the aldosterone level in plasma and urine and a decrease in blood pressure [28].

Selective blood sampling from the adrenal glands and the determination of hormones in it are performed in patients with proven PHA according to a standardized protocol [29]. Principles of preparation for such blood collection are the same as for studies at the first and second stages of diagnosis of PHA.

For differential diagnosis of PHA subtypes, it is recommended to carry out a separate selective collection of blood from the central and segmental veins of the adrenal glands with the further determination of the gradient of concentration of aldosterone and plasma renin at different levels of the venous bed of a separate adrenal gland [30].

The gradient of lateralization, which is the ratio of the aldosterone concentration on the dominant side (the side with its predominant production) to the concentration of aldosterone on the non-dominant side, is also determined. The gradient of lateralization of more than two indicates the unilateral production of aldosterone, whereas the result of less than two indicates an excessive bilateral secretion of the pathologically altered adrenal glands [31, 32].

The sensitivity and specificity of the selective blood collection for determining the lateralization of aldosterone hyperproduction are 95 % and 100% and computed and magnetic resonance tomography – 78% and 75%, respectively [22, 28, 30].

## Conclusions

The laboratory diagnosis of PHA is inherently simple. It allows solving the issues of screening of patients with arterial hypertension, and establishing the presence of PHA, confirmation of the case, delineation of subtypes. At the same time, it should be borne in mind that the results of the study of aldosterone levels depend on many factors. Among them, the conditions of preparing for the study, the method of blood collection, and its treatment are essential. However, when evaluating the blood aldosterone content, the specific features of the clinical manifestations of the disease, the age, and gender of the patients should be taken into account, and the phase of the menstrual cycle in the case of premenopausal women.

## Conflict of Interest

The authors confirm that there are no conflicts of interest.
